# A comprehensive gene expression atlas of sex- and tissue-specificity in the malaria vector, *Anopheles gambiae*

**DOI:** 10.1186/1471-2164-12-296

**Published:** 2011-06-07

**Authors:** Dean A Baker, Tony Nolan, Bettina Fischer, Alex Pinder, Andrea Crisanti, Steven Russell

**Affiliations:** 1 Department of Genetics, University of Cambridge, Downing Street, Cambridge CB1 3QA, UK; 2 Department of Life Sciences, Sir Alexander Fleming Building, Imperial College London, Imperial College Road, London SW7 2AZ, UK; 3 Cambridge Systems Biology Centre, Tennis Court Road, Cambridge CB2 1QR, UK

## Abstract

**Background:**

The mosquito, *Anopheles gambiae*, is the primary vector of human malaria, a disease responsible for millions of deaths each year. To improve strategies for controlling transmission of the causative parasite, *Plasmodium falciparum*, we require a thorough understanding of the developmental mechanisms, physiological processes and evolutionary pressures affecting life-history traits in the mosquito. Identifying genes expressed in particular tissues or involved in specific biological processes is an essential part of this process.

**Results:**

In this study, we present transcription profiles for ~82% of annotated *Anopheles *genes in dissected adult male and female tissues. The sensitivity afforded by examining dissected tissues found gene activity in an additional 20% of the genome that is undetected when using whole-animal samples. The somatic and reproductive tissues we examined each displayed patterns of sexually dimorphic and tissue-specific expression. By comparing expression profiles with *Drosophila melanogaster *we also assessed which genes are well conserved within the Diptera versus those that are more recently evolved.

**Conclusions:**

Our expression atlas and associated publicly available database, the MozAtlas (http://www.tissue-atlas.org), provides information on the relative strength and specificity of gene expression in several somatic and reproductive tissues, isolated from a single strain grown under uniform conditions. The data will serve as a reference for other mosquito researchers by providing a simple method for identifying where genes are expressed in the adult, however, in addition our resource will also provide insights into the evolutionary diversity associated with gene expression levels among species.

## Background

For organisms in which large-scale mutagenic studies are problematic, gene expression catalogues are an important tool for annotating processes on a gene-by-gene basis. In the malarial vector *Anopheles gambiae*, studies have focused on differential expression in males and females [1,2], on samples collected before and after the bloodmeal [2,3] and in dissected tissues such as the midgut [2], salivary glands [4,5], ovaries [2,6], head and carcass [7,8]. However, since these studies often involve different mosquito strains, different experimental platforms and analysis by different statistical methods, comparison among treatments is challenging. Here, we provide a comprehensive expression atlas and associated publicly available database, the MozAtlas (http://www.tissue-atlas.org), cataloguing the relative strength and specificity of gene expression in tissues of male and female mosquitoes using a single genome-wide platform, protocol and analysis.

We employed transcriptional profiling to analyse RNA levels in whole body mosquito samples, eight separate somatic tissues (head, salivary gland, midgut, Malpighian tubules, thoracic and abdominal carcass) and the reproductive tissues (testis, accessory gland, ovary) of males and females separately. In common with the majority of sexually reproducing organisms, *Anopheles *has specialized reproductive traits. Of particular interest is the female-specific activity of blood-feeding, which provides protein for egg development and is a key determinant in *Plasmodium *transmission. In contrast, male mosquitoes feed entirely on sugar, are not adapted for digesting blood and do not transmit malaria. Consequently, those tissues involved in acquiring, ingesting and digesting blood are expected to display substantial sexual dimorphism at the level of gene expression.

In this paper we summarize the functions and sequence level divergence of genes with sexually dimorphic or tissue enriched expression patterns to determine which genes, if any, are rapidly evolving. In addition, by comparing *Anopheles *expression profiles with matched tissues in *Drosophila melanogaster*, we assess evolutionary conservation of expression profiles within the Diptera and identify genes recently evolved in *Anopheles *with tissue specific patterns of expression. Such traits provide ideal candidates for use in population control, where vital or fertility-related genes may be targeted by genetic knockout [9]. With the ongoing development of insect genetics it has become increasingly likely that some pest populations, including mosquitoes, may be controlled with genetic modification [10-15].

## Results

### Gene expression coverage

**Figure 1:**
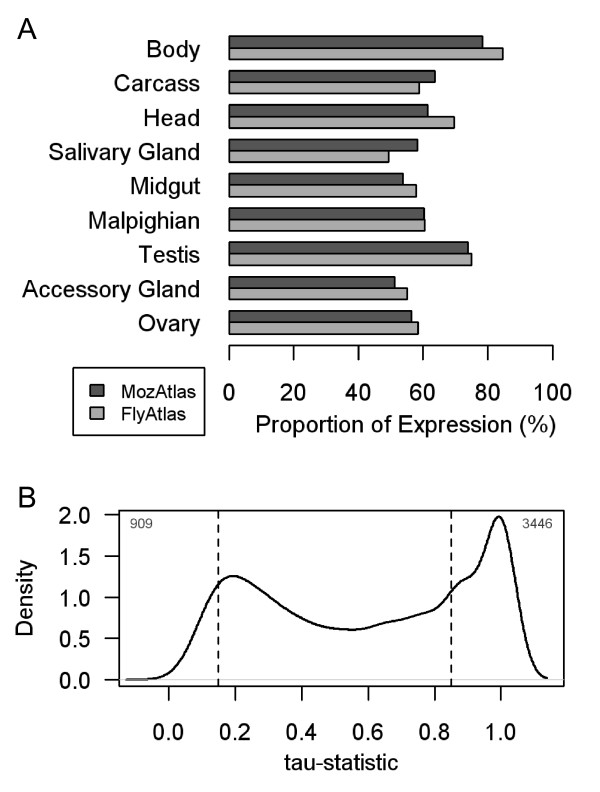
**Global expression coverage**. **(A) **The proportion of probes giving at least 3 out of 4 mismatch calls in either male or female samples for tissue in the MozAtlas and FlyAtlas. **(B) **Tissue breadth. "House-keeping" genes were identified to have a tau-statistic under 0.15 (n = 909), and narrow expression a tau-statistic above 0.85 (n = 3446). Overall, only a third of genes were detected in all tissues.

We have analysed gene expression among *Anopheles *males and females using Affymetrix whole-genome microarrays. The microarray platform contains 16,942 unique *Anopheles *probes corresponding to 10,622 of the annotated protein-coding genes, equating to 82% of the genes in the genome. Female tissues were dissected at 24 hour intervals for a three day period following the blood-meal to provide information on the relative strength and specificity of gene expression in adult mosquito tissues throughout oogenesis. Equivalent male tissues were dissected from siblings in parallel. Array quality was first assessed by calculating the Pearson correlation coefficient between samples. Gene expression was highly similar among replicates (R>= 0.92), indicating that variation in our experiment was low (Additional File 1). While indicating biological replicates are highly consistent, individual-to-individual variation in gene expression will of course be masked by the effect of tissue pooling. After quality control, we detected expression of 10,031 probes corresponding to 7253 unique genes across *Anopheles *tissues. Hierarchical clustering with probe intensities indicates good discrimination of tissues, with expression distributed according to tissue and sex (Additional File 2). The fraction of expressed genes varied from 51% to 74% among samples (Figure 1B). Corresponding *Drosophila *organs analyzed on a similar array platform using the same normalization procedure, found similar levels of relative gene activity. Approximately 20% of *Anopheles *transcripts in dissected samples are absent from whole-body estimates, and only a third of transcripts are recorded across all tissues (Figure 1B).

### Sexually dimorphic gene expression

**Figure 2:**
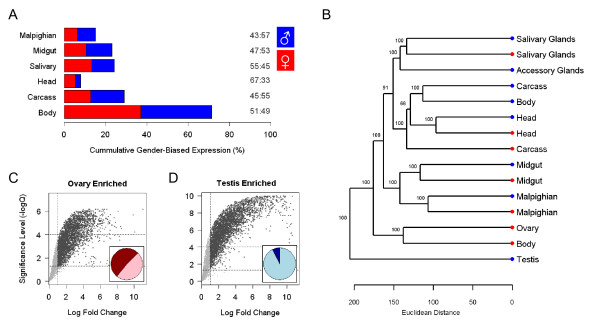
**Sexually dimorphic expression**. **(A) **The proportion of sexually dimorphic expression in each tissue versus total sexual dimorphism. The ratio of female- to male-biased expression is provided (female:male). All ratios deviate significantly from equality (Chi-squared test; P < 0.05). **(B) **Hierarchical clustering of probes among tissues and sex with Euclidean Distance; female (red), male (blue). Branch support was estimated with 10,000 bootstrapped replicates. Expression enrichment against carcass for the **(C) **ovary and **(D) **testis. Significant gonad enrichment is highlighted in dark grey (ANOVA; M > 2; Q < 0.05). Inset figures show the proportion of gonad enrichment which is also sexually dimorphic in whole-body samples, i.e. 51% female-biased (dark red); 7% male-biased expression (dark blue).

To investigate sexually dimorphic expression, a linear model was fit to male and female tissue samples. On the basis of differential expression, we identified probes as either male-biased or female-biased with a 2-fold change of intensity and statistical significance at the Q<0.05 level (Additional File 3). Overall, 54% of genes are sexually dimorphic in at least one organ, including a substantial degree of sex-biased expression in most somatic tissues. Of the 3924 sexually dimorphic genes, 72% are detected in whole-body male and female samples, with the remaining 28% only in dissected tissues (Figure 2A). Each tissue displays a moderate degree of sexual dimorphism, however, by and large, somatic tissues are closely related irrespective of sex when clustered according to expression level (Figure 2B). Thus, each tissue exhibits a specific gene expression profile that is overlaid with sex-specific functions. Sexually dimorphic expression is most skewed in the head, with a high number of female-biased genes detected; in particular we found an over-representation of odorant receptor genes with female biased expression. However, overall, and in all other somatic tissues, there was approximately equal numbers of male- and female-biased genes.

Sexual dimorphism at the gene expression level is associated with different and distinct functional categories (Additional File 4). For example, genes with 'digestion', 'protein metabolism' and 'proteolytic' functions, especially 'serine-type endopeptidase' are over-expressed in the midgut of females. Genes enriched for 'cellular homeostasis', 'ligase activity' and 'transporter activity' are enriched in the female salivary gland, while the malpighian tubules display an over-representation of genes associated with 'ion transportation'. In comparison, male-elevated genes are largely associated with 'carbohydrate metabolic activity', 'ion transporter activity' and 'iron ion binding' within the midgut, salivary gland and Malpighian tubules, as well as the carcass. Ultimately, many of the genes elevated in either sex are of unknown function.

### Tissue specific gene expression

**Figure 3:**
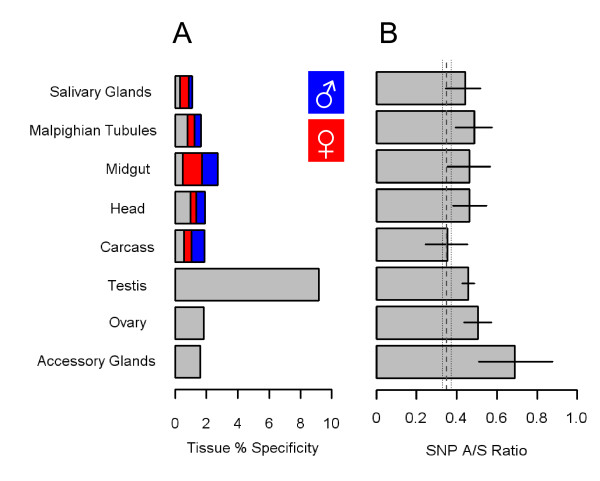
**Tissue-specific expression patterns**. **(A) **Tissue specific transcription was investigated on the basis of probe detection (3 out of 4 mismatch calls). Instances where probes were detected in a single tissue and a single sex are also highlighted: Female (red), male (blue). **(B) **SNP A/S ratio for genes with tissue-specific expression. 95% C.I. was estimated with 10,000 bootstrapped replicates.

In the somatic and reproductive organs examined, a subset of genes showed considerable specificity (Figure 3A). The highest proportion of tissue-specific expression occurs in the testis, where approximately 10% of transcripts are unique. In comparison, ovary specific genes account for ~4% of expression in the tissue, and several of the ovary-specific genes are members of the chorion family. We also found a set of 54 accessory-gland expressed genes, absent from other tissues, representing ~2% of the expression in this tissue (Figure 3A). In common with the *Drosophila *Acps, our *Anopheles *candidates are over-represented in the top 10% of intensity values recorded for the accessory gland (χ^2 ^= 9.45; d.f.= 1; P < 0.003), and many contain secretory domains necessary for transfer to females. Non-reproductive tissues also have a substantial number of genes with specific expression patterns, the majority in a single sex: these are especially prevalent in the midgut, salivary gland and carcass.

Previous studies indicate that genes with restricted expression have elevated rates of sequence divergence amongst related species [16]. We conducted a large-scale survey of SNP A/S ratios using data from dbSNP to determine if such genes were evolving rapidly in *Anopheles *[17]. First, 11,224 genes with at least one coding SNP were collected. In total, ~100,000 coding-region SNPs and 316,043 intronic SNPs were identified, corresponding to SNP densities of 5.6 and 7.19 SNPs, respectively, per 1,000 nucleotides. For our entire dataset, the number of non-synonymous coding SNPs per non-synonymous site (A) was 0.0033, the number of synonymous coding SNPs per synonymous site (S) was 0.0068, and the A/S ratio was 0.49.

SNP A/S estimates of < 1 suggest that most nucleotide substitutions have been eliminated by selection, *i.e*. purifying selection, whereas SNP A/S > 1 indicate that non-synonymous nucleotide substitutions have been maintained, *i.e*. positive selection. As expected, many tissue-specific genes display a higher ratio of A/S SNP ratio than those ubiquitously expressed throughout the organism, *i.e*. fewer non-synonymous mutations have been eliminated by selection and are evolving more rapidly (Figure 3B). For example, genes expressed in reproductive tissues including the testis, ovary and the male accessory gland have the highest rates of sequence divergence within *Anopheles*. Non-reproductive tissues including the head and Malpighian tubules show less deviation, while genes specifically expressed in the salivary gland and midgut have only marginally higher A/S SNP ratios than ubiquitously expressed genes.

### Chromosomal distribution of tissue expression

**Figure 4:**
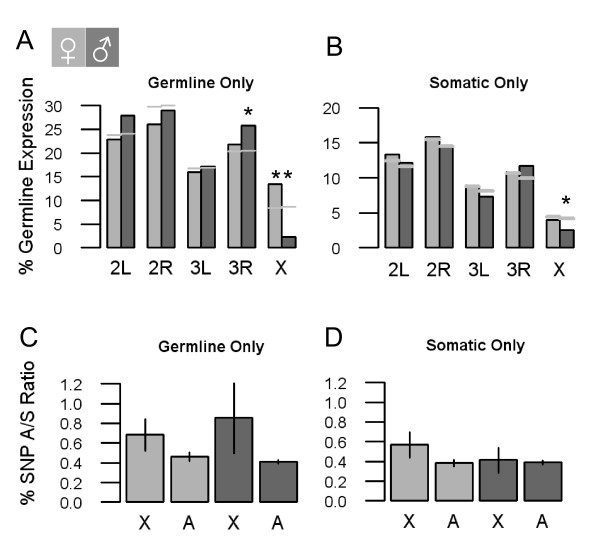
**Gene expression for major chromosome arms**. **(A) **Germline only expression; testis (blue), ovary (red). **(B) **Somatic only expression; male (blue), female (red). **(C) **Germline only *X *vs autosomal SNP A/S ratio. **(D) **Somatic only *X *vs autosomal SNP A/S ratio. (Chi-squared Test; *P < 0.05). Grey bars represent expected proportions. 95% C.I. was estimated with 10,000 bootstrapped replicates.

Across a range of Metazoan species, genes with elevated male expression are non-randomly distributed around the genome [18]. However, in *Anopheles*, previous global estimates of sex-biased expression failed to identify this property [1]. *Anopheles *tissue dissections provide substantially more information about male-specific gene expression than whole-body samples. For example, while a comparison of ovary and carcass expression indicates over half the ovary-enriched genes are female-biased in whole-body samples (Figure 2C), less than 10% of testis-enriched genes are male-biased, largely because they are undetected in whole-body samples (Figure 2D). Our new dataset allowed us to revisit the issue of genome position and expression in reproductive and somatic tissues. We found that genes expressed in the testis, but not the ovary, are under-represented on the *X *chromosome (Figure 4A). In addition, male-biased somatically-expressed genes are also under-represented on the *X *chromosome (Figure 4B). We find that SNP polymorphisms in testis-expressed genes show higher A/S ratios on the *X *chromosome than on the autosomes (χ^2 ^= 26.5 df = 1, P < 2.54 × 10^-7^; Figure 4C). Even though this finding is consistent with the expectation that *X *chromosomes are hostile to testis-expressed genes, the same pattern was not observed with somatic tissues (χ^2 ^= 0.13 df = 1, P = NS; Figure 4D).

### Comparative evolution with Drosophila melanogaster

**Figure 5:**
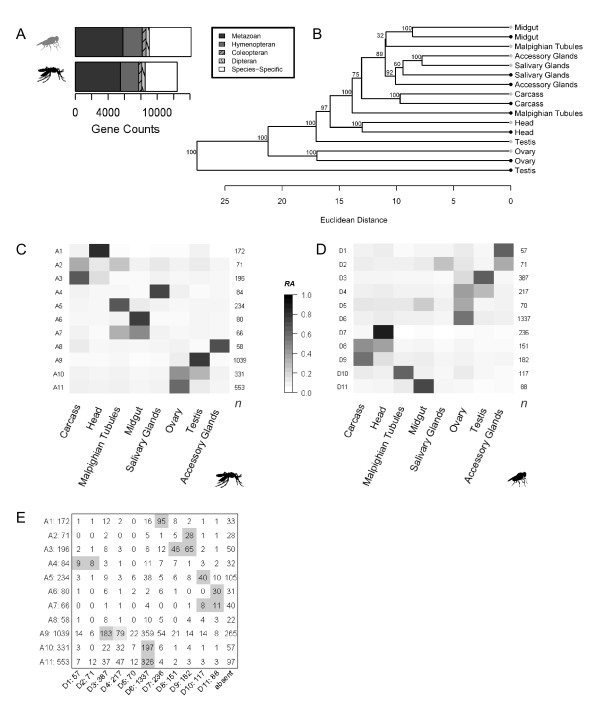
**Orthology Relationships**. **(A) **The oldest common ancestor in gene-families to either a Dipteran, Coleopteran, Hymenopteran or Metazoan ancestor. **(B) **Expression divergence of tissues for one-to-one orthology pairs (n = 4234). Euclidean distance was used to calculate similarity among tissues within and between species. Branch support was estimated with 10,000 bootstrapped replicates. *Drosophila *(grey); *Anopheles *(black). **(C) **Mean expression of *Anopheles *orthologue clusters and **(D) **mean expression of *Drosophila *orthologues clusters. Mean relative expression (RA) level for each cluster according to grayscale. **(E) **The number of overlapping orthologous genes between *Anopheles *and *Drosophila *expression clusters calculated with a hypergeometric probability distribution after multiple correction. Light grey (P < 0.05); Dark grey (P < 0.01).

To estimate evolutionary divergence in tissue expression profiles, orthology relationships in *Drosophila *and *Anopheles *(Insecta: Diptera) were traced back to a common Metazoan ancestor; *Tribolium casteneum *(Insecta: Coleoptera), *Apis melifera *(Insecta: Hymenoptera) or *Caenorhabditis elegans *(Nematoda: Rhabditida) (Figure 5A). From this analysis, we estimate that over half of the genes in the *Anopheles *and *Drosophila *genomes are in 1:1 orthology relationships (n = 6726; Additional File 5); ~95% of which can be traced back to one of the outgroups used in our analysis with the remaining pairs specific to the Dipteran clade. Using these orthologues, we first compared mosquito expression with the same tissues in *Drosophila *[19]. Rather than relying on an absolute measure of gene expression, relative measures of abundance (RA) were calculated for each gene (See Methods). Hierarchical clustering of RA across gene pairs showed that global patterns of expression in homologous organs were often more similar between species than between unrelated tissues within a species (Figure 5B). For some organs (*i.e*. ovary, gut carcass, head), a large proportion of transcriptional variation was conserved between *Anopheles *and *Drosophila*, suggesting that the underlying gene networks have similar functional constraints.

To identify conserved expression signatures underling the above patterns, we used hierarchical clustering with pair-wise correlation coefficients to identify co-expressed genes for each species. We chose clusters with an average similarity of greater than 0.8 and more than 50 genes for further analysis. Overall, 11 clusters meet these criteria in *Anopheles *and *Drosophila*, representing 2884 and 2913 genes respectively (Figure 5C-D; Additional File 6). Adjusting these thresholds, changes the number of groups identified, but were selected to provide a dataset with reasonably sized gene clusters of highly similar expression profiles.

Between species, we evaluated orthologues in each cluster and found several groups with significant overlap (Figure 5E). Typically, co-expression groups are elevated in one or two tissues. For example, a significant number of orthologues are expressed in the head of both the *Anopheles *A1 cluster and the *Drosophila *D7 cluster (Figure 5E). Gene Ontology (GO) annotations for these genes are enriched for 'phototransduction' and 'signal transduction', indicating a close associated with normal physiological functions within head (Table 1). We also found conserved signatures that correspond to expression in the Malpighian tubules, midgut and carcass. A notable exception is that *Anopheles *salivary gland expression (A4), shares most enrichment with *Drosophila *orthologues from the male accessory gland (D1, D2). Overall, the largest clusters are expressed in reproductive tissues (Figure 5C-D). Orthologues with testis expression in *Anopheles*, are spread over a number of *Drosophila *clusters. We further note that a large proportion of orthologues are expressed in the female ovary. Typically, clusters with elevated ovary expression show significant overlap between *Anopheles *and *Drosophila*, and as expected, over-represented GO annotations involve basic cellular processes (Table 1).

**Table 1:** Orthology cluster overlap, tissue expression and enriched gene ontology annotations

*Anopheles*	*Drosophila*	Tissue	GO
A1	D7	Head	phototransduction signal transduction

A2 A3	D9 D8, D9	Carcass	metabolic process cellular respiration

A4	D1, D2	SG, AG	protein folding signal peptide processing

A5 A6 A7	D10 D11 D10, D11	MT Midgut Midgut/MT	transmembrane transport carbohydrate metabolic process

A9	D3, D4	Testis	microtubule-based process spermatogenesis

A10, A11	D6	Ovary	nucleic acid metabolic process oogenesis, cell cycle eggshell formation

SG = salivary gland; AG = Accessory Gland; MT = Malpighian tubules.

Gene ontology significance level (P < 0.01).

### Single-copy and multi-copy gene families

**Figure 6:**
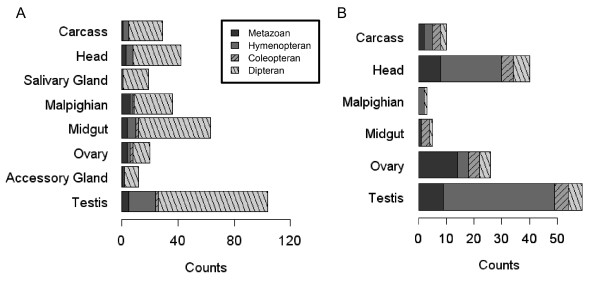
**Gene copies, family origins and tissue expression**. **(A)***Anopheles *gene expansions with restricted expression patterns (n = 325; tau-statistic = 1). **(B) **Single-copy gene-families with narrow spatial expression profiles in both *Drosophila *and *Anopheles *tissues (n = 143; tau-statistic > 0.85).

Our phylogenetic analysis indicated that 5932 families contain a single *Anopheles *gene, whereas another 971 families show evidence of *Anopheles *expansion. In this latter set, duplications with narrow expression patterns (i.e. tau-statistic > 0.85), often arose during the Dipteran split and are most prevalent in the male testis (Figure 6A). However, as well as the testis, a high incidence of duplication events are genes with salivary gland, midgut or Malpighian tubule restricted expression (Figure 6A). Within single-copy families, 143 groups are narrowly expressed in the same *Anopheles *and *Drosophila *tissues (Figure 6B). Such expression is prevalent with head and testis expression, but while these genes might be expected to evolve rapidly, the majority date back to Metazoan and Hymenopteran clades.

### Online MozAtlas Database

For researchers interested in comparing their own experiments to the MozAtlas, we have constructed an online database and web-browser for querying tissue expression in *Anopheles *(http://www.tissue-atlas.org). The single gene query displays tables of normalized expression for each probe and tissue available. In addition, this search displays available orthology relations, a) one-to-one *Drosophila melanogaster *orthologues and corresponding relative gene expression estimates, and b) a gene tree of all mosquito, fly and outgroups within the gene family. We also provide a BLAST and batch searching facilities to output expression values for larger lists of genes that may then be used for further down-stream analysis.

## Discussion

To help improve the functional annotation of the *Anopheles gambiae *genome we have generated the MozAtlas, a unified catalogue of tissue-specific gene expression from a single mosquito strain. In *Drosophila melanogaster*, cataloguing tissue expression patterns has been useful, especially for inferring biological functions, since the majority of genes encoded in the genome are not ubiquitously expressed [19]. As with the fruit fly, *Anopheles *gene expression also exhibits substantial tissue specificity, with only a third of detectably expressed genes found in all tissues. Thus, the MozAtlas is a useful resource for better understanding the mosquito genome, providing direct evidence of genes with tissue restricted expression. Below we highlight the utility of MozAtlas for identifying classes of gene with tissue or sex-biased expression that may be exploited for vector control. Analysis of the MozAtlas also identifies gene expression features that are of interest from an evolutionary perspective, revealing both highly conserved and species-specific aspects of insect biology. Of particular interest, given that malaria parasites are only transmitted through female mosquitoes, we separately catalogued gene expression for each tissue in males and females, thus providing both tissue and sex-specific views of gene expression in the adult.

A major finding from our analysis is the substantial degree of sexually dimorphic gene expression we find at the tissue level: more than half of the genes for which we detect expression exhibit sexual dimorphism in terms of expression level. The head, in particular, has a significantly higher number of female-biased genes and of these, odorant receptors are significantly over-represented (Additional File 4). When searching for a blood-meal, female mosquitoes are attracted to odours emitted by humans, a behaviour mediated by receptors in the antennal sensilla [7]. This activity is not exhibited by males, who feed entirely on nectar, and we presume that the female elevated expression of odorant binding molecules reflect this biology. The identification of molecules associated with female-specific aspects of odorant detection may provide targets for controlling malaria transmission [20].

We identified other sexually dimorphic expression signatures that appear to be associated with female characteristics, in particular, adaptation to hematophagy. For example in the female salivary gland we found an over-representation of genes with protein and lipid catabolic activity, ion transport and cellular homostasis functions. We suggest that these reflect the fact that, in females, the salivary gland produces compounds to disarm host hemostatic and immune responses, thus allowing mosquitoes to take a blood-meal. Similarly, many proteins found in the midgut are only synthesized by blood-feeding females [3,21]: numerous digestive and proteolytic molecules implicated in blood digestion were identified as female elevated in our analysis.

In contrast, elevated male gene activity is largely associated with carbohydrate metabolism and ion transport activity. Since male mosquitoes feed entirely on sugar, these results were not surprising. However, somewhat more novel is that iron binding molecules are up-regulated in males. While in female mosquitoes iron is especially important for egg development and is strongly influenced by blood-feeding [22], iron metabolism has diverse physiological and developmental roles [23]. Although females obtain iron from the blood meal, the sugar diet of males may necessitate more efficient iron uptake and up-regulation of genes that encode iron binding functions.

In both somatic and reproductive tissues, we identified genes with considerable specificity. Tightly controlled, tissue-specific expression is of interest for understanding the basic biology of a species, and is likely to be key in the development of next generation insect control agents. For example, genes uniquely expressed in particular tissues could be targets for inducing sterility or providing regulatory elements to drive localised expression of transgenes. In this respect, the highest proportion of *Anopheles *tissue-specific expression is in the testis, with approximately 10% of transcription uniquely detected in this tissue. Testis specific expression of genes with important roles in spermatogenesis, sperm competition or sperm-egg interactions present a set of targets with potential for inducing male sterility.

After mating, *Anopheles *females undergo distinct behavioural and physiological changes due to the transfer of both sperm and proteins produced in the male accessory glands [24]: proteins secreted by males and passed to females in seminal fluid could provide a route for altering female fertility. Via specific expression profiling of accessory glands we have identified a new set of potential *Anopheles *Acp genes that will enable further investigation of sexual conflict within the mosquito. Sexual antagonism between males and females may be expected to cause rapid Acp sequence evolution [25]. We find that among tissue-specific genes, those expressed in the accessory gland have a higher A/S ratio than in many tissues, including the testis. Slower evolutionary rates in the *Anopheles *testis might be explained, in part, by their mating behaviour: in polyandrous insects genes involved in spermatogenesis are often under strong positive selection as a result of post-copulatory male-male competition [25], whereas these pressures in the testis are expected to be absent from the largely monandrous *Anopheles *mosquitoes [26].

Genes with ovary specific expression provide potential targets for inducing female sterility in mosquitoes given that they are closely associated with egg formation. Chorion components of the fruit fly eggshell, for example, provide the embryo with protection from the physical environment, and disrupting their function causes female sterility [27]. Recently, proteomic techniques have identified *Anopheles *eggshell constituents, several of which we find to be specifically expressed in the ovary, making them favourable candidates for use in population control [28].

In terms of genome structure, we show that genes with male-biased expression are non-randomly distributed around the *Anopheles *genome. Two mechanisms have been proposed to explain the disparity in chromosomal distribution of male expressed genes. First, during spermatogenesis the *X *chromosome of males becomes inactivated: since few testis genes are expressed post-meiotically, evidence suggests that chromosomal inactivation has promoted autosomal duplication events from *X*-linked genes [18,29,30]. There is compelling evidence that *X*-linked inactivation also occurs in nematodes [31] and mammals [32], however, an under-representation of male-biased somatically-expressed genes on the *X *chromosome indicates that other forces are also at work. Second, since males only have one *X *chromosome, polymorphisms beneficial to one sex may arise that are detrimental to the other sex. Such antagonistic sexual selection may eventually lead to sequence changes and demasculinization of the *X *chromosome [33], and consistent with this expectation, genes on the *Anopheles X *chromosome have less sequence polymorphism than on the autosomes.

Identifying expression divergence within and between closely-related species provides important insights into the selective pressures underlying gene regulation [34,35]. The opportunity to compare divergence between *Drosophila *and *Anopheles*, separated by some 250 million years of evolution, allows us to explore gene and tissue evolution over a considerable time scale. We find that expression similarity in one-to-one orthologues of the midgut, head, carcass and ovary expressed genes is well conserved in the *Diptera *and, as expected, genes in conserved co-expression clusters perform integral physiological functions.

In contrast, tissues such as the testis, often show considerable transcriptional variation between closely related species [36,37]. It's been proposed that testis gene regulation plays a critical role in the initial formation of reproductive isolation [38]. In addition to the *Anopheles *testis, expression in other tissues is also highly divergent: for example, expression in the Malpighian tubules is largely not conserved between *Anopheles *and *Drosophila*. As an organ with a key role in detoxification and osmoregulation, this divergence may reflect fundamental differences in the diet of each insect [39]. In addition, salivary gland and male accessory gland expression cluster within rather than between species, evidence for a bout of simultaneous evolution since the last common ancestor was shared. Indeed, no significant co-expression was detected between species, indicating that secretory organ functions have diverged during the Dipteran split.

Recent *Anopheles *gene duplications are often expressed in the testis and, in *Drosophila*, extreme expansions also have spermatogenesis-related functions [40]. As well as the testis, other tissues display narrow expression profiles of recent origin in *Anopheles*. Certainly, the blood meal imposes a range of challenges on the digestive system of mosquitoes and, in part, explains a predominance of gene duplications with salivary gland, Malpighian tubule or midgut expression. Even between members of the same mosquito subgenera, salivary proteins can diverge rapidly over time [41]: our data suggests that this evolutionary pattern may also be common in Malpighian tubule proteins and, to a lesser extent, proteins within the midgut. However, specifically expressed genes in large families do not necessarily highlight unique functions, since homologues may perform the same or similar functions in a larger set of tissues. Gene families with single members are of interest for identifying unique processes, given that closely related homologues are not found within the genome. Narrowly expressed single-copy families were detected dating back to Metazoan and Hymenopteran clades, perhaps accompanying the emergence of differentiated organs. It will be of considerable interest for insect control programs to determine whether such proteins perform integral functions in their specific tissues, given that as single copies they should perform unique roles within the organism.

## Conclusions

We have generated a tissue and sex-specific gene expression atlas for *Anopheles gambiae *and used it to explore mosquito biology related to reproduction, feeding and gene evolution. Given that *Anopheles *is the major vector of one of the world's most debilitating diseases, our dataset provides an important reference for other mosquito researchers wishing to explore potential roles for genes of interest. Of particular importance is the identification of uniquely expressed genes that may serve as tissue-specific drivers in transgenic constructs or potential knockout targets in the next generation of insect control agents.

## Methods

### RNA collections and microarray platform

Male and female mosquito siblings were separated at pupation and allowed to emerge into separate cages to prevent mating. 3-day old, non-mated females were blood-fed and female tissues were dissected at 24 hour intervals for a three day period following the blood-meal. Equivalent male tissues were dissected from age-matched siblings in parallel. Dissections were carried out in phosphate-buffered saline using dissecting needles and a 28 gauge needle to cleanly separate connected tissues from each other. 'Midgut' samples were dissected clear of the foregut, hindgut and malphigian tubules to include the anterior midgut and stomach. 'Head' samples were produced by severing at the neck and include brain, eyes, cuticle and some fat body. 'Ovary' samples include both ovaries and the common oviduct. 'Salivary gland' samples include the salivary duct, lateral lobes and median lobe. Salivary glands were rinsed extensively in PBS to remove the majority of fat body associated with the glands. 'Carcass' includes the thoracic and abdominal carcass and all tissues therein excluding those tissues individually described in the MozAtlas. Dissected tissues were placed immediately in Trizol to minimize the impact of dissection on the transcriptome. For each of four biological replicates, tissues were pooled from a minimum of 10 mosquitoes dissected at each time point. For each tissue and sex, an equal quantity of total RNA was pooled from three time points sampled after the blood-meal to obtain gene expression estimates throughout oogenesis (24, 48, 72 hrs). Each RNA sample (50 ng) was subsequently amplified in two cycle cDNA target labelling to generate biotinylated cRNA probes for hybridization on to Affymetrix microarrays [42].

### Estimates of gene expression

Oligonucleotide probes and genes were mapped to AgamP3 genome assembly. Unless otherwise stated, datasets were analyzed with the R statistical programming language using programs maintained as part of the Bioconductor suite [43]. In addition to microarray datasets for *Anopheles*, matching tissues obtained from the *Drosophila *FlyAtlas were re-analyzed with the same normalization procedure (GEO: GSE1690; GSE7763). Intensity values between arrays were first standardized within tissues for each species separately using the robust multi-array analysis package [44,45]. The expression presence and absence calls were assessed with the signal to noise ratio of the perfect match and mismatch probes provided on Affymetrix arrays. Probes were used in further analysis only if they were deemed to be present in at least three tissue replicates. All estimates of differential expression were adjusted for multiple testing using the false discovery rate method [46]. Array data has been submitted to the Gene Expression Omnibus under GSE21689.

### Sexual dimorphism and tissue specificity

Sexual dimorphism was determined with a linear model of gene expression fit to male and female samples for each tissue as implemented in the LIMMA library [47]. On the basis of differential expression, we subsequently identified probes as either male-biased or female-biased where there was a significant 2-fold change of intensity in one sex, in addition to statistical significance at the Q< 0.05 level (Additional File 3). Two measures of tissue specificity were also calculated: probe detection and tissue expression breadth. Probes were deemed tissue-specific if at least 3 out of 4 mismatch calls were found, but only in a single tissue and sex. In comparison, tissue breadth was measured by normalizing against maximal expression to generate the tau-statistic [48]. The resulting tau-statistic falls within the range of 0 to 1, in which higher values indicate greater tissue-biased expression. *Anopheles *and *Drosophila *Gene Ontology annotations (Biological Process, Molecular Function, Cellular Component) and the enrichment of functions were determined using FlyMine with a 1% false discovery rate for multiple testing correction [49].

### SNP polymorphism

When sequences are available for multiple individual in a species, the ratio of observed non-synonymous mutation rate (A) to the synonymous mutation rate (S) can be utilized as an estimate of the selective pressure. To estimate sequence polymorphism within *Anopheles *we conducted a large-scale survey of dbSNP [17]. While it is not possible to measure selective constraint on individual proteins directly using this approach, it has been demonstrated that when a group of genes are measured together, estimates of variation are robust and in good agreement with A/S for divergence [50].

### Expression divergence

Since microarray platforms were designed separately for *Drosophila *and *Anopheles*, probes have different affinities to their target RNAs, making the normalization of orthology expression between chips difficult. In order to compare tissue expression profiles between species, each gene was represented as a vector of relative expression abundance (RA) across the sampled tissues to avoid over-estimating divergence based on absolute expression intensity. Where genes are represented by multiple probes, the maximum intensity value recorded in each tissue was used for subsequent analysis. Since the FlyAtlas does not have separate samples for males and females, we combined male and female samples in the MozAtlas to make comparisons between species. Hierarchical clustering of orthologues was performed with measures of RA within and between species. For gene-wise clustering, we used Pearson correlation coefficient as the distance measure and defined similarity between clusters using average-linkage clustering. Co-regulated genes were defined as any group with an average similarity of greater than 0.8 that also contained more than 50 genes. Among species clusters, orthologue overlap was subsequently investigated with a hypergeometric probability distribution to determine enrichment.

### Orthology classification

DNA and protein sequences were obtained for *D. melanogaster *and *A. gambiae *(Ensembl v50) [51], *Tribolium casteneum *(Version 3; BeetleBase) [52], *Apis melifera *(Version 2; BeeBase) [53] and *Caenorhabditis elegans *(ws160; Ensembl v50) [51]. One-to-one orthology relationships were determined using Inparanoid with default parameters, we selected the longest available translation for each annotated protein [54]. Best reciprocal hits between species were grouped together into broader gene-families, and the sequences aligned with MUSCLE [55]. Tree topologies were subsequently reconstructed with both dS (synonymous substitution rate), dN (nonsynonymous substitution rate), nucleotide and protein distance measures using TreeBest [56,57]. From back-translation of protein alignments, TreeBest creates a consensus tree by merging the results of neighbour joining and maximum likelihood (ML) trees. By default, ML trees based on protein alignment are built under the WAG model, while ML tree based on DNA are built under the HKY model, which models non-uniform base composition and transition/transversion rate bias [58]. Orthology relationships are described as one-to-one, one-to-many and many-to-many gene relationships.

## Authors' contributions

DAB conceived, analysed and designed the study, performed microarray experiments with BF, and wrote the paper. TN and AP reared the mosquitoes and dissected the tissue samples. TN, SR and AC participated in the design of this study and helped write the paper. AC and SR raised the funding. All authors read and approved the final document.

## Acknowledgements

This study was funded by a Grant from the Foundation for the National Institutes of Health through the Grand Challenges in Global Health initiative.

## Supplementary Material

Additional file 1 **Figure S1 - Correlation of gene expression between replicate samples**. Click here for file

Additional file 2 **Figure S2 - Hierarchical clustering of probe intensity**. Click here for file

Additional file 3 **Figure S3 - Sexual dimorphism of tissue gene expression**. Click here for file

Additional file 4 **Table S1 - Gene Ontology annotation of sexual dimorphic gene expression**. Click here for file

Additional file 5 Table S2 - One-to-one orthology relationships and gene expression. Click here for file

Additional file 6 **Table S3 - Cluster annotation of one-to-one orthology relationships**. Click here for file

## References

[B1] Hahn MW Lanzaro GC Female-biased gene expression in the malaria mosquito *Anopheles gambiae* Curr Biol 2005 15 R192 193 10.1016/j.cub.2005.03.005 15797007

[B2] Marinotti O Genome-wide analysis of gene expression in adult *Anopheles gambiae* Insect Mol Biol 2006 15 1 12 10.1111/j.1365-2583.2006.00610.x 16469063

[B3] Dana AN Hong YS Kern MK Hillenmeyer ME Harker BW Lobo NF Hogan JR Romans P Collins FH Gene expression patterns associated with blood-feeding in the malaria mosquito *Anopheles gambiae* BMC Genomics 2005 6 5 10.1186/1471-2164-6-5 15651988 PMC546002

[B4] Arcà B Lombardo F Valenzuela JG Francischetti IM Marinotti O Coluzzi M Ribeiro JM An updated catalogue of salivary gland transcripts in the adult female mosquito, *Anopheles gambiae* J Exp Biol 2005 208 3971 86 10.1242/jeb.01849 16215223

[B5] Calvo E Pham VM Lombardo F Arcà B Ribeiro JM The sialotranscriptome of adult male Anopheles gambiae mosquitoes Insect Biochem Mol Biol 2006 36 570 5 10.1016/j.ibmb.2006.04.005 16835022

[B6] Koutsos AC Blass C Meister S Schmidt S MacCallum RM Soares MB Collins FH Benes V Zdobnov E Kafatos FC Christophides GK Life cycle transcriptome of the malaria mosquito Anopheles gambiae and comparison with the fruitfly Drosophila melanogaster Proc Natl Acad Sci USA 2007 104 11304 9 10.1073/pnas.0703988104 17563388 PMC2040894

[B7] Biessmann H Nguyen QK Le D Walter MF Microarray-based survey of a subset of putative olfactory genes in the mosquito Anopheles gambiae Insect Mol Biol 2005 14 575 89 10.1111/j.1365-2583.2005.00590.x 16313558

[B8] Iatrou K Biessmann H Sex-biased expression of odorant receptors in antennae and palps of the African malaria vector Anopheles gambiae Insect Biochem Mol Biol 2008 38 268 74 10.1016/j.ibmb.2007.11.008 18207086 PMC2247438

[B9] Burt A Site-specific selfish genes as tools for the control and genetic engineering of natural populations Proc Biol Sci 2003 270 921 928 10.1098/rspb.2002.2319 12803906 PMC1691325

[B10] Heinrich J Scott M A repressible female-specific lethal genetic system for making transgenic insect strains suitable for a sterile-release program Proc Natl Acad Sci USA 2000 97 8229 8232 10.1073/pnas.140142697 10890889 PMC26929

[B11] Thomas DD Donnelly CA Wood RJ Alphey LS Insect population control using a dominant, repressible, lethal genetic system Science 2000 287 2474 2476 10.1126/science.287.5462.2474 10741964

[B12] Franz AW Sanchez-Vargas I Adelman ZN Blair CD Beaty BJ James AA Olson KE Engineering RNA interference-based resistance to dengue virus type 2 in genetically modified *Aedes aegypti* Proc Natl Acad Sci USA 2006 103 4198 4203 10.1073/pnas.0600479103 16537508 PMC1449670

[B13] Phuc HK Andreasen MH Burton RS Vass C Epton MJ Pape G Fu G Condon KC Scaife S Donnelly CA Late-acting dominant lethal genetic systems and mosquito control BMC Biol 2007 5 11 10.1186/1741-7007-5-11 17374148 PMC1865532

[B14] Windbichler N Papathanos PA Catteruccia F Ranson H Burt A Crisanti A Homing endonuclease mediated gene targeting in *Anopheles gambiae *cells and embryos Nucleic Acids Res 2007 35 5922 5933 10.1093/nar/gkm632 17726053 PMC2034484

[B15] Fu G Lees RS Nimmo D Aw D Jin L Gray P Berendonk TU White-Cooper H Scaife S Kim Phuc H Female-specific flightless phenotype for mosquito control Proc Natl Acad Sci USA 2010 107 4550 4 10.1073/pnas.1000251107 20176967 PMC2826341

[B16] Larracuente AM Sackton TB Greenberg AJ Wong A Singh ND Sturgill D Zhang Y Oliver B Clark AG Evolution of protein-coding genes in *Drosophila* Trends Genet 2008 24 114 123 10.1016/j.tig.2007.12.001 18249460

[B17] Sherry ST dbSNP: the NCBI database of genetic variation Nucleic Acids Res 2001 29 308 311 10.1093/nar/29.1.308 11125122 PMC29783

[B18] Sturgill D Zhang Y Parisi M Oliver B Demasculinization of x chromosomes in the *Drosophila *genus Nature 2007 450 238 241 10.1038/nature06330 17994090 PMC2386140

[B19] Chintapalli VR Wang J Dow JA Using FlyAtlas to identify better *Drosophila melanogaster *models of human disease Nat Genet 2007 39 715 720 10.1038/ng2049 17534367

[B20] Carey AF Wang G Su C Zwiebel LJ Carlson JR Odorant reception in the malaria mosquito Anopheles gambiae Nature 2010 464 66 71 10.1038/nature08834 20130575 PMC2833235

[B21] Warr E Aguilar R Dong Y Mahairaki V Dimopoulos G Spatial and sex-specific dissection of the *Anopheles gambiae *midgut transcriptome BMC Genomics 2007 8 37 10.1186/1471-2164-8-37 17261194 PMC1804276

[B22] Dunkov B Georgieva T Insect iron binding proteins: insights from the genomes Insect Biochem Mol Biol 2006 36 300 309 10.1016/j.ibmb.2006.01.007 16551544

[B23] Nichol H Law JH Winzerling JJ Iron metabolism in insects Annu Rev Entomol 2002 47 535 559 10.1146/annurev.ento.47.091201.145237 11729084

[B24] Rogers DW Whitten MM Thailayil J Soichot J Levashina EA Catteruccia F Molecular and cellular components of the mating machinery in *Anopheles gambiae *females Proc Natl Acad Sci USA 2008 105 19390 19395 10.1073/pnas.0809723105 19036921 PMC2614771

[B25] Haerty W Jagadeeshan S Kulathinal RJ Wong A Ravi Ram K Sirot LK Levesque L Artieri CG Wolfner MF Civetta A Evolution in the fast lane: rapidly evolving sex-related genes in *Drosophila* Genetics 2007 177 1321 1335 10.1534/genetics.107.078865 18039869 PMC2147986

[B26] Tripet F Touré YT Dolo G Lanzaro GC Frequency of multiple inseminations in field-collected *Anopheles gambiae *females revealed by DNA analysis of transferred sperm Am J Trop Med Hyg 2003 68 1 5 12556139

[B27] Galanopoulos VK Orr W Szabad J Kafatos FC Genetic analysis of chorion formation in *Drosophila melanogaster*: I. The effects of one somatic-specific and seven germ-line-specific mutations Dev Genet 1989 10 87 97 10.1002/dvg.1020100204 2499437

[B28] Amenya DA Chou W Li J Yan G Gershon PD James AA Marinotti O Proteomics reveals novel components of the *Anopheles *gambiae eggshell J Insect Physiol 2010 56 1414 1419 10.1016/j.jinsphys.2010.04.013 20433845 PMC2918668

[B29] Vibranovski MD Zhang Y Long M General gene movement off the × chromosome in the *Drosophila *genus Genome Res 2009 19 897 903 10.1101/gr.088609.108 19251740 PMC2675978

[B30] Kemkemer C Hense W Parsch J Fine-scale analysis of × chromosome inactivation in the male germline of Drosophila melanogaster Mol Biol Evol 2010 in press 10.1093/molbev/msq355 21193490

[B31] Bean CJ Schaner CE Kelly WG Meiotic pairing and imprinted × chromatin assembly in *Caenorhabditis elegans* Nat Genet 2004 36 100 105 10.1038/ng1283 14702046 PMC4098868

[B32] Khil PP Smirnova NA Romanienko PJ Camerini-Otero RD The mouse × chromosome is enriched for sex-biased genes not subject to selection by meiotic sex chromosome inactivation Nat Genet 2004 36 642 646 10.1038/ng1368 15156144

[B33] Wu CI Xu EY Sexual antagonism and × inactivation--the SAXI hypothesis Trends Genet 2003 19 243 247 10.1016/S0168-9525(03)00058-1 12711214

[B34] Khaitovich P Hellmann I Enard W Nowick K Leinweber M Franz H Weiss G Lachmann M P ääbo S Parallel patterns of evolution in the genomes and transcriptomes of humans and chimpanzees Science 2005 309 1850 1854 10.1126/science.1108296 16141373

[B35] Zhang Y Sturgill D Parisi M Kumar S Oliver B Constraint and turnover in sex-biased gene expression in the genus *Drosophila* Nature 2007 450 233 237 10.1038/nature06323 17994089 PMC2386141

[B36] Ranz JM Castillo-Davis CI Meiklejohn CD Hartl DL 2003, Sex-dependent gene expression and evolution of the *Drosophila *transcriptome Science 2003 300 1742 1755 10.1126/science.1085881 12805547

[B37] Meiklejohn CD Parsch J Ranz JM Hartl DL Rapid evolution of male-biased gene expression in *Drosophila* Proc Natl Acad Sci USA 2003 100 9894 9899 10.1073/pnas.1630690100 12907700 PMC188344

[B38] Voolstra C Tautz D Farbrother P Eichinger L Harr B Contrasting evolution of expression differences in the testis between species and subspecies of the house mouse Genome Res 2007 17 42 49 17038563 10.1101/gr.5683806 PMC1716265

[B39] Wang J Kean L Yang J Allan AK Davies SA Herzyk P Dow JA Function-informed transcriptome analysis of *Drosophila *renal tubule Genome Biol 2004 5 R69 10.1186/gb-2004-5-9-r69 15345053 PMC522876

[B40] Hahn MW Han MV Han SG Gene family evolution across 12 *Drosophila *genomes PLoS Genet 2007 3 e197 10.1371/journal.pgen.0030197 17997610 PMC2065885

[B41] Arcà B Lombardo F Francischetti IM Pham VM Mestres-Simon M Andersen JF Ribeiro JM An insight into the sialome of the adult female mosquito *Aedes albopictus* Insect Biochem Mol Biol 2007 37 107 27 10.1016/j.ibmb.2006.10.007 17244540

[B42] Affymetrix GeneChip Expression Analysis Technical Manual. 701021 Rev. 5 2004 Santa Clara, CA, Affymetrix

[B43] Gentleman RC Carey VJ Bates DM Bolstad B Dettling M Dudoit S Ellis B Gautier L Ge Y Gentry J Hornik K Bioconductor: open software development for computational biology and bioinformatics Genome Biol 2004 5 R80 10.1186/gb-2004-5-10-r80 15461798 PMC545600

[B44] Irizarry RA Exploration, normalization, and summaries of high density oligonucleotide array probe level data Biostatistics 2003 4 249 264 10.1093/biostatistics/4.2.249 12925520

[B45] Wu Z Irizarry RA Gentleman R Martinez-Murillo F Spencer F A Model-Based Background Adjustment for Oligonucleotide Expression Arrays J Am Stat Assoc 2004 99 909 10.1198/016214504000000683

[B46] Hochberg Y Controlling the false discovery rate: a practical and powerful approach to multiple testing J Royal Stat Soc B 1995 57 289 300

[B47] Smyth GK Linear models and empirical bayes methods for assessing differential expression in microarray experiments Stat Appl Genet Mol Biol 2004 3 Article 3 10.2202/1544-6115.1027 16646809

[B48] Yanai I Benjamin H Shmoish M Chalifa-Caspi V Shklar M Ophir R Bar-Even A Horn-Saban S Safran M Domany E Genome-wide midrange transcription profiles reveal expression level relationships in human tissue specification Bioinformatics 2005 21 650 659 10.1093/bioinformatics/bti042 15388519

[B49] Lyne R Smith R Rutherford K Wakeling M Varley A Guillier F Janssens H Ji W Mclaren P North P FlyMine: an integrated database for *Drosophila *and *Anopheles *genomics Genome Biol 2007 8 R129 10.1186/gb-2007-8-7-r129 17615057 PMC2323218

[B50] Liu J Zhang Y Lei X Zhang Z Natural selection of protein structural and functional properties: a single nucleotide polymorphism perspective Genome Biol 2008 9 R69 10.1186/gb-2008-9-4-r69 18397526 PMC2643940

[B51] Flicek P Aken BL Ballester B Beal K Bragin E Brent S Chen Y Clapham P Coates G Fairley S Ensembl's 10th year Nucleic Acids Res 2010 38 D557 562 10.1093/nar/gkp972 19906699 PMC2808936

[B52] Wang L Wang S Li Y Paradesi MS Brown SJ BeetleBase: the model organism database for *Tribolium castaneum* Nucleic Acids Res 2007 35 D476 479 10.1093/nar/gkl776 17090595 PMC1669707

[B53] Elsik CG Worley KC Zhang L Milshina NV Jiang H Reese JT Childs KL Venkatraman A Dickens CM Weinstock GM Community annotation: procedures, protocols, and supporting tools Genome Res 2006 16 1329 1333 10.1101/gr.5580606 17065605

[B54] O'Brien KP Remm M Sonnhammer EL Inparanoid A Comprehensive Database of Eukaryotic Orthologs NAR 2005 33 D476 D480 15608241 10.1093/nar/gki107 PMC540061

[B55] Edgar RC MUSCLE: a multiple sequence alignment method with reduced time and space complexity BMC Bioinformatics 2004 5 113 10.1186/1471-2105-5-113 15318951 PMC517706

[B56] TreeSoft: Softwares for Phylogenetic Trees http://treesoft.sourceforge.net/treebest.shtml

[B57] Ruan J TreeFam: Update Nucleic Acids Res 2008 36 D735 D740 18056084 10.1093/nar/gkm1005 PMC2238856

[B58] Guindon S Lethiec F Duroux P Gascuel O PHYML Online-a web server for fast maximum likelihood-based phylogenetic inference Nucleic Acids Res 2005 33 W557 W559 10.1093/nar/gki352 15980534 PMC1160113

