# Supplementary material for: A comprehensive gene expression atlas of sex- and tissue-specificity in the malaria vector, Anopheles gambiae
Source: BMC Genomics. 2011 Jun 7;12:296. doi: 10.1186/1471-2164-12-296 (PMC3129592; doi:10.1186/1471-2164-12-296)

Supplementary Fig. 1. Correlation between replicates. For each tissue and sex, normalized intensity values of replicate hybridizations were plotted against each other and the corresponding Pearson's correlation co-efficient calculated. All treatments display high correlation, indicating low variation between replicates and excellent reproducibility.

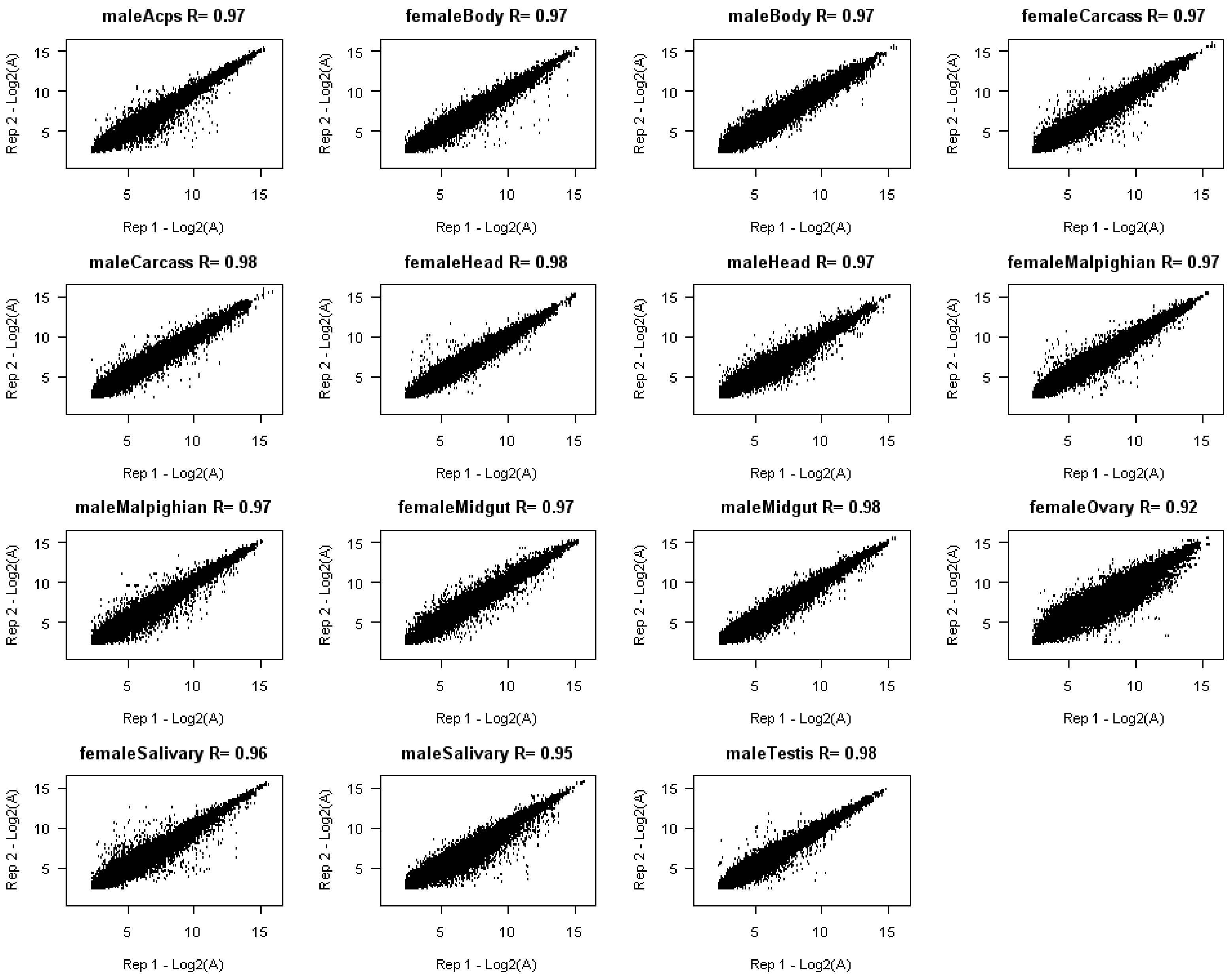

Supplement: Additional file 1 — Figure S1 - Correlation of gene expression between replicate samples. [file 1471-2164-12-296-S1.PDF]
