# Supplementary material for: A comprehensive gene expression atlas of sex- and tissue-specificity in the malaria vector, Anopheles gambiae
Source: BMC Genomics. 2011 Jun 7;12:296. doi: 10.1186/1471-2164-12-296 (PMC3129592; doi:10.1186/1471-2164-12-296)

Supplementary Fig.2. Hierarchical clustering of probe intensity among tissue replicates as measured by Euclidean Distance. Branch support was estimated with 10,000 bootstrapped replicates.

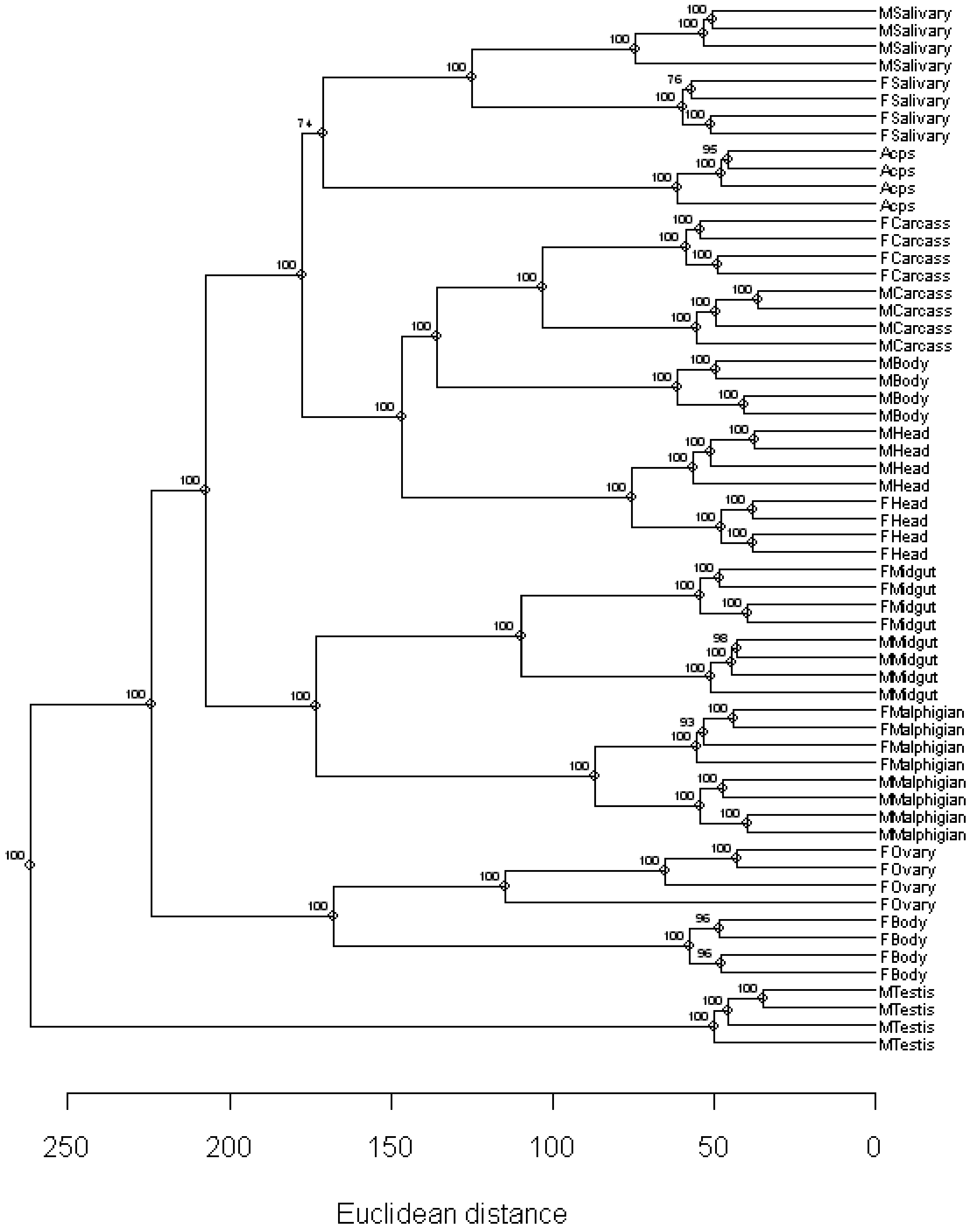

Supplement: Additional file 2 — Figure S2 - Hierarchical clustering of probe intensity. [file 1471-2164-12-296-S2.PDF]
