# Supplementary material for: A comprehensive gene expression atlas of sex- and tissue-specificity in the malaria vector, Anopheles gambiae
Source: BMC Genomics. 2011 Jun 7;12:296. doi: 10.1186/1471-2164-12-296 (PMC3129592; doi:10.1186/1471-2164-12-296)

Supplementary Fig. 3. Sexual dimorphism. A linear model was fit to male and female gene expression separately for each tissue. On the basis of differential expression, probes were identified as either male-biased or female-biased if there was a significant 2-fold elevation of intensity in one gender (M>2; Q<0.05); a) body, b) carcass, c) head, d) salivary gland, e) midgut f) malpighian tubules.

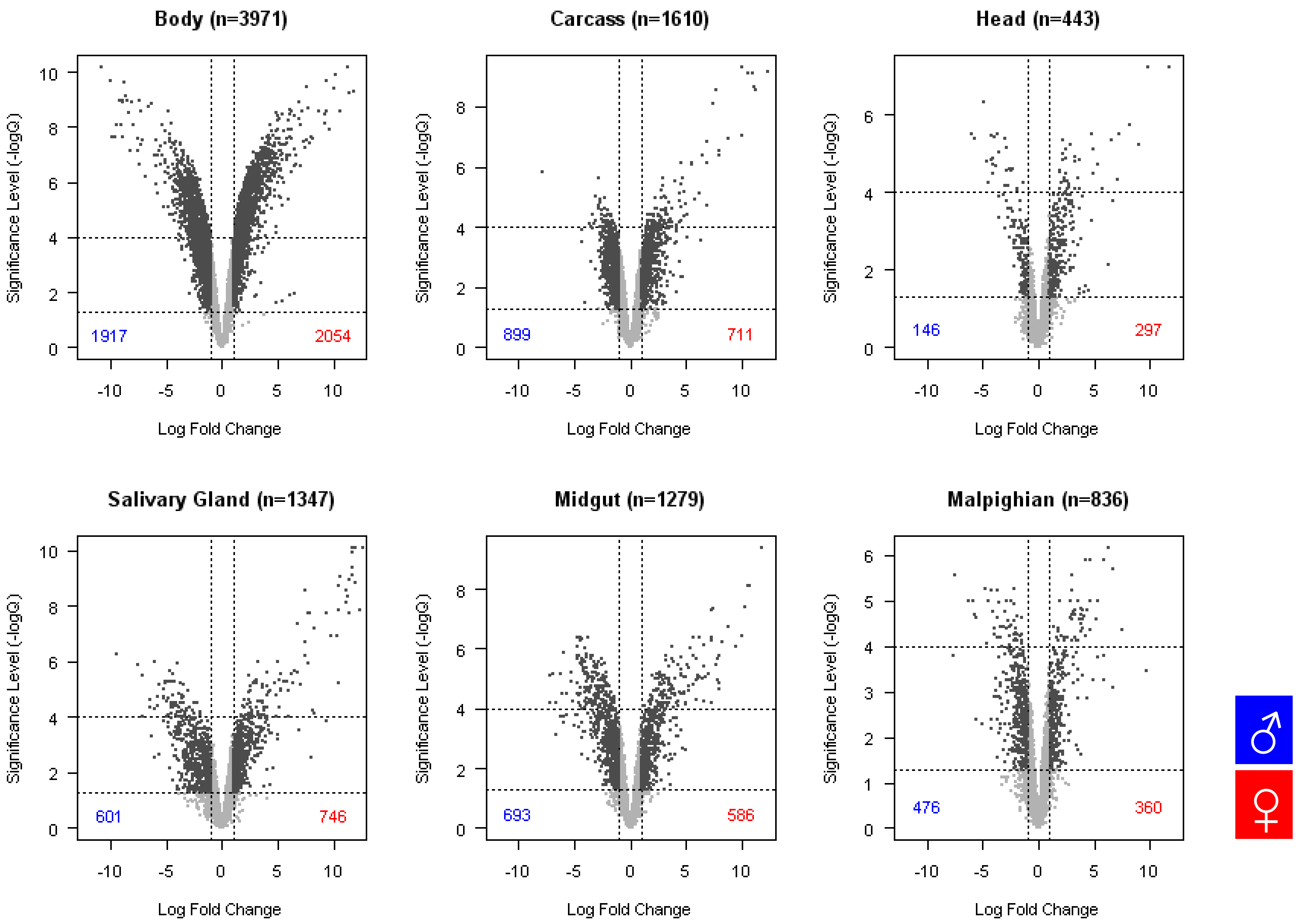

Supplement: Additional file 3 — Figure S3 - Sexual dimorphism of tissue gene expression. [file 1471-2164-12-296-S3.PDF]
